# Low-Cost and Paper-Based Micro-Electromechanical Systems Sensor for the Vibration Monitoring of Shield Cutters

**DOI:** 10.3390/s24165349

**Published:** 2024-08-19

**Authors:** Yazhou Zhang, Xinggang Li, Jiangfan Fu, Linpeng Liu, Changchao Zhang, Ji’an Duan

**Affiliations:** 1 State Key Laboratory of Precision Manufacturing for Extreme Service Performance, College of Mechanical and Electrical Engineering, Central South University, Changsha 410083, China; asiazhang@126.com (Y.Z.); 233712172@csu.edu.cn (J.F.); duanjian@csu.edu.cn (J.D.); 2 China Railway 14th Bureau Group Co., Ltd., Jinan 250101, China; xingganglee@126.com; 3 Key Laboratory of Bionic Engineering, Ministry of Education, Jilin University, Changchun 130022, China; changchaozhang@jlu.edu.cn

**Keywords:** vibration sensor, paper-based, graphite, low cost, easy fabrication

## Abstract

Vibration sensors are widely used in many fields like industry, agriculture, military, medicine, environment, etc. However, due to the speedy upgrading, most sensors composed of rigid or even toxic materials cause pollution to the environment and give rise to an increased amount of electronic waste. To meet the requirement of green electronics, biodegradable materials are advocated to be used to develop vibration sensors. Herein, a vibration sensor is reported based on a strategy of pencil-drawing graphite on paper. Specifically, a repeated pencil-drawing process is carried out on paper with a zigzag-shaped framework and parallel microgrooves, to form a graphite coating, thus serving as a functional conductive layer for electromechanical signal conversion. To enhance the sensor’s sensitivity to vibration, a mass is loaded in the center of the paper, so that higher oscillation amplitude could happen under vibrational excitation. In so doing, the paper-based sensor can respond to vibrations with a wide frequency range from 5 Hz to 1 kHz, and vibrations with a maximum acceleration of 10 g. The results demonstrate that the sensor can not only be utilized for monitoring vibrations generated by the knuckle-knocking of plastic plates or objects falling down but also can be used to detect vibration in areas such as the shield cut head to assess the working conditions of machinery. The paper-based MEMS vibration sensor exhibits merits like easy fabrication, low cost, and being environmentally friendly, which indicates its great application potential in vibration monitoring fields.

## 1. Introduction

The motion of objects or the operation of machines with rotating/vibrating parts always generate vibrations. Similar to acoustics, vibrations are one of the main kinds of communicational signals that animals or humans use to obtain information from the environment or individuals [1,2,3,4]. Therefore, vibration signals can be an indicator to reflect some information about the hosted sources [5,6,7]. To decipher the information contained in the vibration signal, vibration sensors are widely used to detect vibrations so that we can access the exact conditions of the target by analyzing the vibration characteristics like frequency, amplitude, speed, and acceleration in real-time, from the feedback of the sensors [5,8,9,10,11]. Currently, vibration sensors made of rigid materials like silicone, metal, ceramic, and sapphire are commonly used in practical industrial applications [12,13,14,15,16]. The fabrication of commercial vibration sensors is complicated and high-cost, and often requires advanced techniques like photolithography, wet etching, electronic beam evaporation, and ion implantation [14,17,18,19]. Diversified application scenarios lead to great differences in the required sensors’ performances, but usually sensor manufacturers are unwilling to accept customized products with small batches, because customization takes too long and has a significant cost, as well as the sales not being guaranteed. Therefore, the sensors used in industry are generally not the most suitable ones but have to compromise the existing products of sensor manufacturers. Therefore, how to develop vibration sensors with low cost and easy fabrication is of great significance for the wide use of vibration sensors and customized performances at any time.

The rapid development of sensors brings huge promotion for many fields like agriculture, military, medicine, the environment, etc. [20,21,22,23,24,25]. However, speedy upgrading along with the rapid proliferation of these devices is giving rise to an increased amount of electronic waste (E-waste) around the world [26,27]. Currently, the E-waste produced every year significantly increases the demand for landfill space and causes environmental burdens at the same time due to the sensor containing a lot of metals, non-metals, or even toxic materials which are difficult to refine and recycle materials [28,29]. Therefore, the usage of renewable or biodegradable materials to manufacture electronics has become a popular research trend to address these challenges. Recently, biocompatible or biodegradable materials like silk, cellulose, starch, chitin, and chitosan, etc., have attracted wide attention to be used to develop sensors [26,30,31,32,33]. Among these materials, paper, with many merits including renewability, biodegradability, and low cost, shows great potential as an ideal substrate for fabricating eco-friendly sensors. Most reported paper-based sensors are used to detect strain [34,35,36,37,38], pressure [39,40,41,42,43,44], humidity [45,46], temperature, or even electrochemical signals [47,48]. For example, Qi et al. prepared a mulberry paper-based graphene strain sensor with high mechanical strength and large area via the Meyer rod-coating technique, achieving a high durability and great mechanical resistance of 1.13 MPa [36]. Chen et al. developed a flexible pressure sensor based on printing paper and carbonized crepe paper, which showed great sensitivity, fast response time (<30 ms), and a low detection limit of ~0.9 Pa [42]. 

In this work, we report on a paper-based vibration sensor with a similar harmonic oscillator architecture to traditional MEMS sensors. Specifically, a zigzag-shaped photo paper framework was engraved by laser, and parallel microgrooves were fabricated on the surface of the photo paper. To make the microgrooves work like the piezoresistors of traditional MEMS sensors, pencil drawing on paper was repeatedly deployed to cover the microgrooves with graphite, forming a functional conductive layer for electromechanical signal conversion. Meanwhile, to enhance the oscillating amplitude of the zigzag-shaped photo paper under vibration excitation and obtain a larger electrical output, a mass was added in the center of the paper framework. The results demonstrate that the paper-based sensor has a wide frequency bandwidth ranging from 5 Hz to 1 kHz, and a maximum acceleration sensing range of up to 10 g. The developed sensor not only can be utilized for monitoring vibrations generated by knuckle-knocking tables or objects falling down but also can be used to detect vibrations in areas such as the shield cut head to assess the working conditions of machinery. The paper-based MEMS vibration sensor exhibits merits like easy fabrication, low cost, and being environmentally friendly, which indicates its great application potential in vibration monitoring fields.

## 2. Materials and Methods

### 2.1. Materials

Photo paper (230 g/m^2^, Kodak, Eastman Kodak Co., NewYork, NY, USA) and pencils (12B) were bought from a local stationer. Silver paste was purchased from the Shanghai Julong Electronic Technology Co., Ltd., China. Copper paste was bought from Dongguan Hengchuang Adhesive Products Co., Ltd., China. PI adhesive tape was provided by Shenzhen Huijia Adhesive Products Co., Ltd., China.

### 2.2. Fabrication of Vibration Sensor

**Figure 1:**
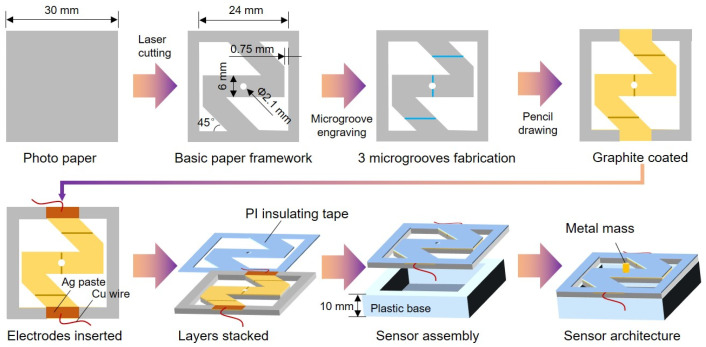
Schematic fabrication and the architecture of the paper-based MEMS vibration sensor.

A square piece of paper with a side length of 30 mm was cut by a femtosecond laser. Then, the squared paper was engraved with a specially designed pattern. A zig-zag-shaped structure was formed with a width of 6 mm, while an angle of 45° was formed between the paper base and the inclined part of the zig-zag-shaped framework. The width of the paper base was 3 mm, and a spacing of 0.75 mm was controlled between the paper base and the zig-zag-shaped framework. A hole with a diameter of 2.1 mm was inserted in the center of the zig-zag-shaped framework. After PI tape was used to encapsulate the graphite-coated paper, a hole with a diameter of 2 mm was inserted in the center of the PI tape. By doing this, a metal mass with a weight of 300 mg was installed in the center, insulated with the graphite layer. Detailed design parameters can be seen in Figure 1. As for the microgrooves, they were designed to be perpendicular to the current direction, and there were only 3 of them (Figure 1). The coated graphite on paper was controlled at a mass density of 0.02 g/cm^2^ and the initial resistance of the sensor was ~40 KΩ. Then, silver paste and copper foil were used to create electrodes for the sensor at the initial and terminal ends of the zig-zag-shaped framework. Finally, the paper/graphite/PI hybrid membrane was fixed on a special plastic base. 

### 2.3. Characteristics of the Vibration Sensor

The surface geometry of the sensor was characterized by an ultra-depth field microscope (VHX5000, Keyence, Japan). Different types and frequencies of signals were generated by the test system, including the shaker (SA-JZ002, Wuxi Shiao Technology Ltd., China), the power amplifier (SP-PA003, Wuxi Shiao Technology Ltd., China), and the signal generator (DG1022Z, RIGOL, China). The resistance signals of the vibration sensor were read by a digit multimeter (DAQ6510, KEITHLEY, America). The acceleration of the input vibration and the voltage signals at the sensor terminals were captured by the dynamic data collector (SA1808A2, Shiao, China).

## 3. Results and Discussion

### 3.1. The Design of Paper-Based Vibration Sensor

Figure 1 illustrates the architecture of the paper-based vibration sensor. It is well known that photo paper has a high gloss side and a very rough side. Generally, photo paper has higher breaking strength and better wrinkle resistance than other commercial papers like printing paper and tissue paper. For the substrates of developing vibration sensors, paper that is too soft or hard is not suitable. This is because soft paper has poor elastic properties which make it hard to recover after the acting force disappears, while hard paper has great elastic properties which make it hard to deform and is insensitive to mechanical vibrations. So, we used photo paper as the substrate of the vibration sensor due to it having appropriate mechanical properties like strength and elasticity. We engraved a zig-zag-shaped framework within a squared photo paper, forming a central symmetry but making the zig-zag-shaped framework oscillate up and down freely. In other words, two symmetrical ends of the zig-zag-shaped framework are set to be connected to the base of the squared photo paper, while the other two ends are not connected. Attributed to this special design, the zig-zag-shaped framework can oscillate in the vertical direction without deviation to the X or Y axis due to the gravity highly located in the center. 

To make the zig-zag-shaped framework transfer its mechanical deformations into electric signals, conductive materials should be fabricated on the paper surface. We used a pencil to draw on paper to form a graphite layer on the paper surface. However, microstructures should be introduced, otherwise the resistance of the graphite layer will not change significantly under vibrations. Many reported works have demonstrated that microstructures like micro- or nanodomes, pyramids, pores, wrinkles, cracks, etc., are beneficial to improving the sensor’s performances such as sensitivity, working range, detection limitation, and so on [49,50]. It is known that good mechanical properties of substrates are the foundation of real applications for sensors. Photo paper is a type of paper that has a stronger tensile strength than other daily used papers like tissue paper, printing paper, packaging paper, etc. However, while it is hard for photo paper to fabricate out-of-plane structures on its surface, it is comparatively feasible to process suitable microstructures in a subtractive manufacturing way. Therefore, we intended to fabricate microgrooves on the paper’s surface by femtosecond laser, which has been verified as useful in enhancing the electrical output in our previous works [51,52]. 

Thus, the direction of microgrooves is designed to be perpendicular to the current flow, as shown in Figure 1. After the microgrooves are etched on the surface of photo paper, a graphite layer is formed to cover the paper surface by drawing with a pencil. Undoubtedly, the microgrooves are also filled with graphite. Then, we used Ag paste and Cu wires to fabricate electrodes, followed by a lasered PI insulating tape to encapsulate and protect the conductive graphite layer. Finally, the multilayered membrane is installed on a plastic base with a central cavity. To make the sensitive stacked membrane easier to oscillate up and down under vibrations, we set and fixed a mass in the center of the membrane to enhance inertia, which is similar to the commercial vibration sensor made by PVDF with a mass (Figure 1). 

### 3.2. Working Mechanism of Paper-Based Vibration Sensor

**Figure 2:**
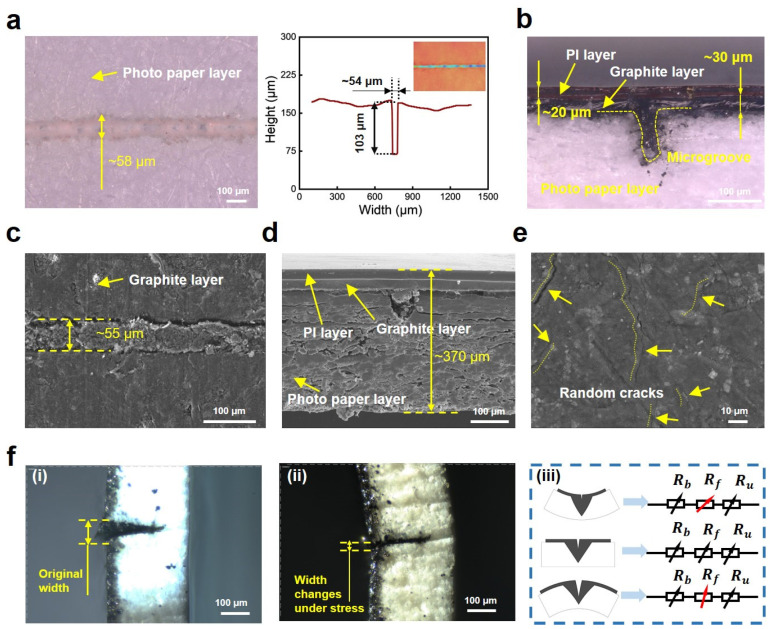
Morphology characterization of the paper-based MEMS sensor and its working mechanism. (**a**) Optical image of the paper surface after etching by femtosecond laser. And the size of groove is characterized by an ultra-depth field microscope showing the width and depth of microgroove. (**b**) Optical image showing the cross-sectional shape of the sensor’s surface after graphite coating and PI tape encapsulated. (**c**) SEM image of the top surface after graphite coating. (**d**) SEM image of the cross-sectional geometry showing the interfaces of different layered materials. (**e**) SEM image of the surface after graphite is drawn on paper, showing the micro/nano cracks generated under stress. (**f**) The working mechanism of the sensor. (**i**) Optical image of the microgroove with original width. (**ii**) Optical image of the microgroove whose width changes under stress. (**iii**) The impact of the microgroove on the resistance of the sensor.

The surface and cross-sectional geometry of the photo paper before and after pencil drawing are observed. We designed a number of three microgrooves on the paper surface, two of them are parallel to the X-axis and the other one is parallel to the Y-axis. Obviously, the optical images in Figure 2a show the lasered microgroove has a width of ~58 μm after the microgroove etching of the femtosecond laser. After the pencil is drawn on the paper surface repeatedly, a graphite layer with a thickness of ~30 μm is formed, followed by a PI-encapsulated layer with a thickness of ~20 μm (Figure 2b). The coated graphite layer almost covers the microgrooves whereas clear boundaries can be observed. The SEM image in Figure 2c shows the graphite fills the microgrooves, and the width of the microgroove after being graphite-coated is 55 μm approximately. We also observed the cross-sectional geometry using a scanning electron microscope. As shown in Figure 2d, after the sensor is frozen by liquid nitrogen and undergoes brittle bending stress to form a fracture surface, there are clear interfacial boundaries due to the sensor consisting of three-layered materials. From top to bottom, there are PI tape, graphite, and photo paper layers in sequence, and the sensor has a thickness of ~370 μm. Since the coated graphite layer has poor stretchability, many micro/nano cracks are generated on the surface of the graphite layer when the sensor suffers tensile stress, as shown in Figure 2e. Although micro/nano cracks are undesirable in materials since they are harmful and easily lead to failure, it has been demonstrated that they can enhance the output electric signal amplitudes of sensors so that many reported works are based on the micro/nano cracks to develop advanced sensors with high sensitivity [38,53]. 

After the geometry characterization of the sensor, we further analyzed its working mechanism to show how the sensor transforms mechanical deformations into electric signals. As shown in Figure 2f, when the sensor is unloaded, the widths of the grooves are initially consistent with the original, designed width. However, when the sensor is subjected to vibrations, the sensitive membrane oscillates up and down under inertia. Thus, the sensitive membrane suffers bending strains and the widths of the microgrooves change with the oscillation. For example, when the sensitive membrane oscillates down, the top half part of the sensitive membrane (namely, the side with grooves) bears compressive stress, and then the widths of the microgrooves decrease when compared to the original widths. Similarly, when the sensitive membrane oscillates up, tensile stress acts on the top half part, causing the widths of the microgrooves to increase. Since the direction of the microgrooves is perpendicular to the direction of current, thus the deformation of the graphite-coated microgrooves is important which helps construct a variable conductive network for the sensor to respond to mechanical stimuli with electric output. When the widths of the microgrooves decrease, it results in a larger contact area between the graphite coating on the sidewalls of the groove and the graphite filled in the groove. Then, an increased number of conducting paths makes the sensor’s resistance drop. On the contrary, the increase in the widths of microgrooves would make the contact area decrease, and then the number of conducting paths drops, causing the sensor’s resistance to grow. At the same time, cracks generated on the coated graphite surface also play an important role in the resistance change, which relies on the change in conductive paths [53].

### 3.3. Sensing Performance of Paper-Based Vibration Sensor

**Figure 3:**
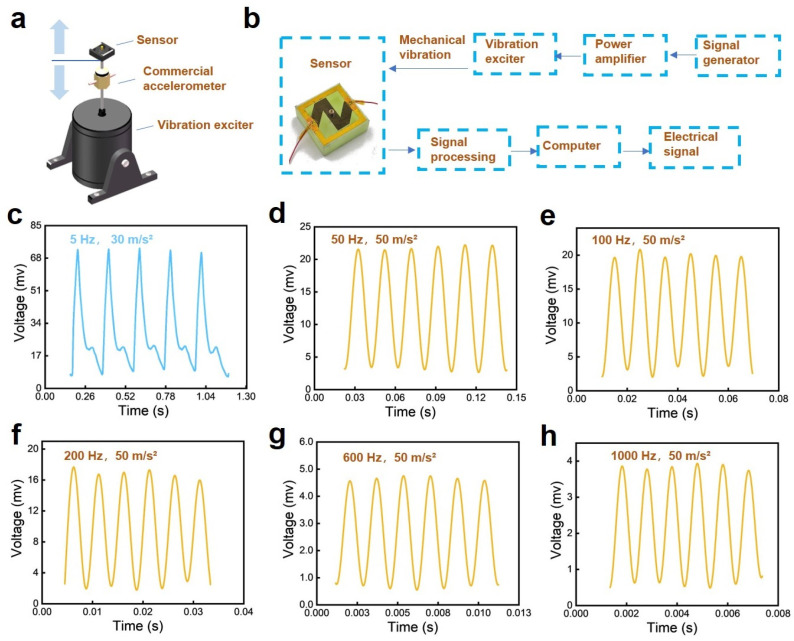
The response of the paper-based MEMS sensor to vibrations with different frequencies. (**a**) Illustration of the test device used to evaluate the sensor’s electric response to vibration stimuli. (**b**) A flow chart of the whole test system, which corresponds to the experiments. (**c**–**h**) The sensor’s responses to applied vibration with a frequency of 5 Hz, 50 Hz, 100 Hz, 200 Hz, 600 Hz, and 1000 Hz.

Figure 3a exhibits that the sensor, as well as a commercial accelerometer, is installed on the output shaft of a vibration exciter. When the vibration exciter is driven by input vibration waveform signals, the output shaft drives the commercial accelerometer and the developed sensor to perform vibrating motions up and down. Figure 3b shows the whole test system, which principally includes a signal generator, a power amplifier, a vibration exciter, and data acquisition/analysis equipment. Meanwhile, the sensor is connected to a 5 V circuit in series with a fixed 43k Ohm resistor, to transfer the resistance signal into a voltage signal so that the data acquisition device with high-frequency sampling can collect data. Figure 3c–h indicate the electric response of the paper-based sensor to vibration stimuli with frequencies of 5 Hz, 50 Hz, 100 Hz, 200 Hz, 600 Hz, and 1000 Hz. It is apparent that the sensor has regular responses to the excited vibrations, and the periods of the peaks in these plots are consistent with the corresponding period of the input vibrations. All the peaks in each plot are kept at the same level. As the frequency of vibration increases, the maximum output voltage amplitude corresponding to the peaks decreases. This is because the displacement of the output shaft of the vibration exciter drops when the frequency of vibration increases. 

**Figure 4:**
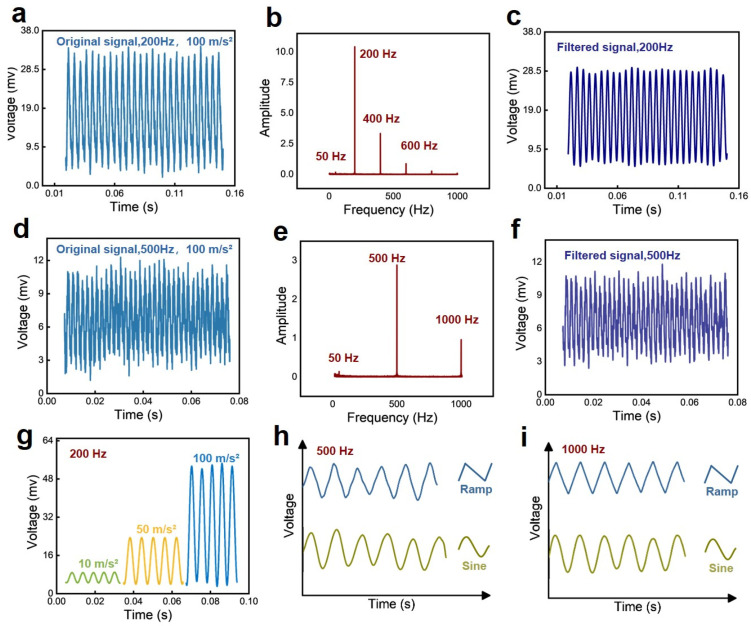
(**a**) The original signal of the sensor at a vibration frequency of 200 Hz. (**b**) The Fourier transform of the voltage signal and (**c**) the voltage signal after filtering out 50 Hz noise from (**a**). (**d**) The original signal of the sensor at a vibration frequency of 200 Hz. (**e**) The Fourier transform of the voltage signal and (**f**) the voltage signal after filtering out 50 Hz noise from (**d**). (**g**) The relative voltage changes of the sensor responded to vibrations with different accelerations but with the same frequency of 200 Hz. (**h**,**i**) Real-time response of the sensor to different vibration waveforms but with the same frequency of 500 Hz, and 1000 Hz.

To further demonstrate that the developed sensor can recognize the frequency of input vibration, we used the methods of fast Fourier transform (FFT) to process the collected voltage signals. Figure 4a shows the real-time voltage signal of the sensor under an applied vibration at a frequency of 200 Hz. Figure 4b,c show the FFT result and the data processed result after filtering out the 50 Hz noise from the environment. It can be seen that the frequency-domain signal output by the paper-based sensor are mainly concentrated on 50 Hz, 200 Hz, 400 Hz, and 600 Hz. Among them, 50 Hz represents the environmental interference, and the maximum peak at 200 Hz is the dominant frequency of the output signal, while 400 and 600 Hz refer to the harmonic output from the sensor during the vibration process. Obviously, the dominant frequency signal output by the paper-based sensor distinguishes itself in that it has a much larger magnitude than its harmonics. After filtering out the 50 Hz noise from the environment, the actual voltage signal output by the sensor is shown in Figure 4c. Similar results can be obtained after analyzing the voltage signal of the sensor at a frequency of 500 Hz (Figure 4d). The peak of the harmonic signal concentrating on 1000 Hz is much smaller than the peak of the dominant frequency signal (Figure 4e), and the periodic sinusoidal signal can be obtained after 50 Hz filtering (Figure 4f). Figure 4g reflects the sensor’s responses to vibrations at a fixed frequency of 200 Hz but different accelerations at 1 g, 5 g, and 10 g. The sensor has a maximum voltage amplitude of ~10 mv when the acceleration is 1 g. The maximum voltage amplitude increases to ~55 mv when the acceleration reaches 10 g. In addition, the fabricated sensor also shows recognition capability for different input vibration waveforms such as ramp and sine under different frequencies, as shown in Figure 4h,i.

**Figure 5:**
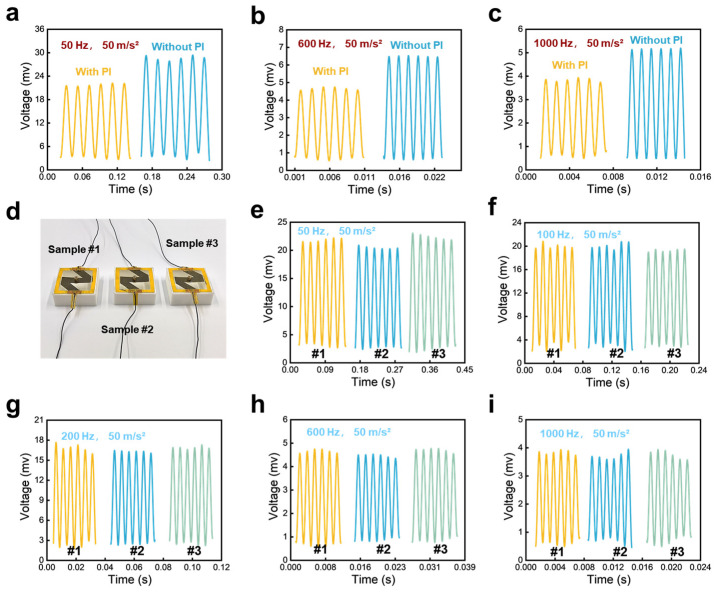
(**a**–**c**) The impact of PI encapsulation on the sensing properties of sensors. (**d**) Three sensors fabricated in the same way, they are named sample #1, sample #2, and sample #3, respectively. (**e**–**i**) Real-time response of the sensor to different vibrations with a fixed acceleration of 50 m/s^2^ but different frequencies at 50 Hz, 100 Hz, 200 Hz, 600 Hz, and 1000 Hz.

In addition, we also study the impact of PI encapsulation on the sensing performance of sensors. We compare the responses of sensors both before and after packaging (i.e., w/o, w/the PI film) to vibrations at frequencies of 50 Hz, 600 Hz, and 1000 Hz (Figure 5a–c). It is evident that the sensors without PI packaging exhibit higher response amplitudes compared to those with PI packaging. This difference is likely because the PI film, when attached to the sensor, restricts the deformation of the sensor’s sensing layer, thereby reducing the extent of micro-channel deformation and consequently decreasing the voltage variation in the packaged sensors. Although PI encapsulation slightly reduces the amplitude of the sensor’s response to applied vibrations, it significantly enhances protection for the sensing layer. For example, scratching the surface with the edge of a paper towel or a glass plate causes noticeable damage to the sensing layer, which can be entirely avoided with the use of PI-encapsulated material. 

In addition, we also tested the sensor’s repeatability and reproducibility to determine that the fabrication of the sensor is reliable. We fabricated three sensors in the same way and compare these sensors’ responses under different mechanical vibrations. As shown in Figure 5d–i, when vibrations with a fixed acceleration of 50 m/s^2^ but different frequencies at 50 Hz, 100 Hz, 200 Hz, 600 Hz, and 1000 Hz are loaded to the paper-based sensors, the responses from the three sensors are highly consistent, demonstrating that the manufacturing method can be used to develop sensors in a mass.

### 3.4. Applications of Paper-Based Vibration Sensor

**Figure 6:**
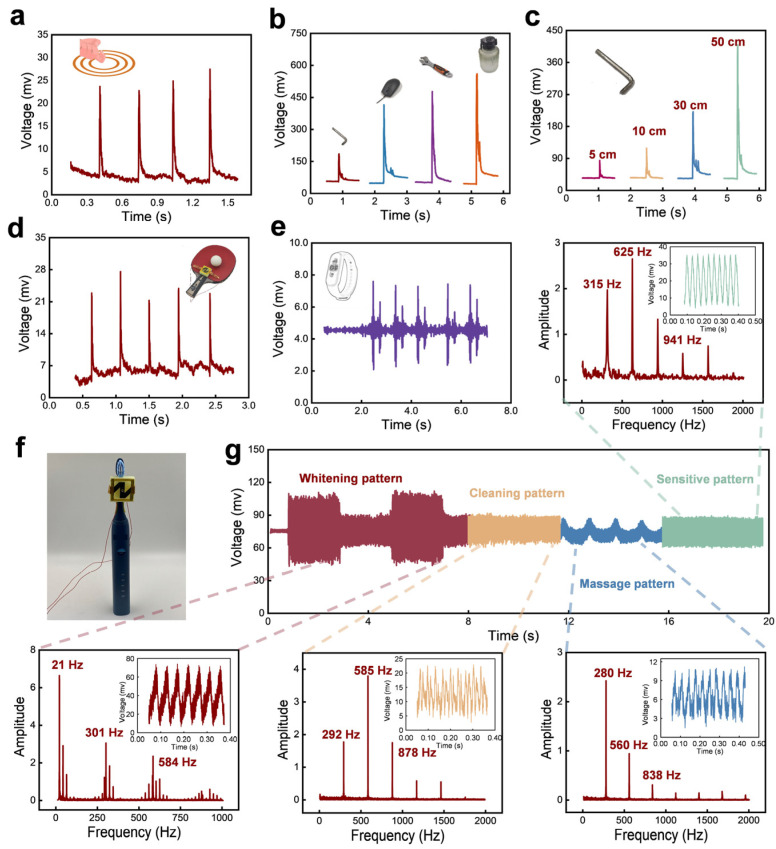
The applications of the fabricated paper-based MEMS sensor used to detect vibration stimuli. (**a**) Output voltage of the paper-based MEMS sensor when a volunteer’s knuckle knocked the table at a distance of 10 cm away from the sensor. (**b**) Output voltage of the paper-based MEMS sensor when different objects generated vibration stimuli at a distance of 10 cm away from the sensor. (**c**) Output voltage of the paper-based MEMS sensor when a hex wrench fell from heights of 5 cm, 10 cm, 30 cm, and 50 cm, above the spot on the table at a distance of 10 cm away from the sensor. (**d**) Response of the sensor to detect the vibration generated by the bounce of a ping-pong ball on a table tennis racket. (**e**) Response of the sensor to detect the vibration generated by the alarm clock of the sports bracelet. (**f**) Photograph of the fabricated sensor stacked on a vibrating toothbrush. (**g**) Real-time electric signal feedback of the sensor, and the FFT analysis results of the output signals for different operating modes of the toothbrush.

When it comes to practical applications, we used the developed paper-based sensor to detect vibrations generated in our daily lives. For example, we fixed the sensor on a plastic plate and knocked the plate by knuckle at a distance of ~10 cm. Significant peaks can be found in the real-time recorded plot of the voltage signal, as shown in Figure 6a. Different items from light to heavy, including a hex wrench, mouse, plier, and plastic water bottle, fell from a height of 30 cm, above the spot on the plate at a distance of ~10 cm away from the sensor. The heavier the dropping item, the larger the peak value (Figure 6b). Figure 6c shows the response of the sensor to the hex wrench falling from different heights (5 cm to 50 cm) but above the falling spot at a distance of ~10 cm away from the sensor. Undoubtedly, as the falling height increases, the peak value of the sensor’s output signal grows, indicating the generated vibration intensity also augments. 

In addition, the sensor is also used to detect the vibrations generated by a ping-pong ball bouncing continuously in the central position of the table tennis racket (Figure 6d), as well as the alarm clock vibration of the power bracelet (Figure 6e), showing that the sensor can serve as wearable electronics to monitor human sports or health. To verify the sensor can be also used to detect high-frequency vibration in daily life, the sensor is installed on the brush head of the electric toothbrush (Figure 6f). The electric toothbrush has five operating modes, which in turn are cleaning, whitening, polishing, sensitive, and massage modes. Figure 6g gives the responses of the developed sensor under the five different operating modes. When the electric toothbrush works in a cleaning mode, the FFT analysis result shows that the first dominant frequency occurs at 292 Hz, while the maximum peak frequency is 585 Hz. When the electric toothbrush changes to whitening mode, the first dominant frequency coincides with the maximum peak frequency at 21 Hz and the real-time waveforms of the sensor are regular. Similarly, when the electric toothbrush works in the other three modes, the output signals from the developed sensor behave with clear frequency responses, each of them has a first dominant frequency larger than 200 Hz, showing the sensor has the potential to detect high-frequency vibrations in some engineering fields. 

**Figure 7:**
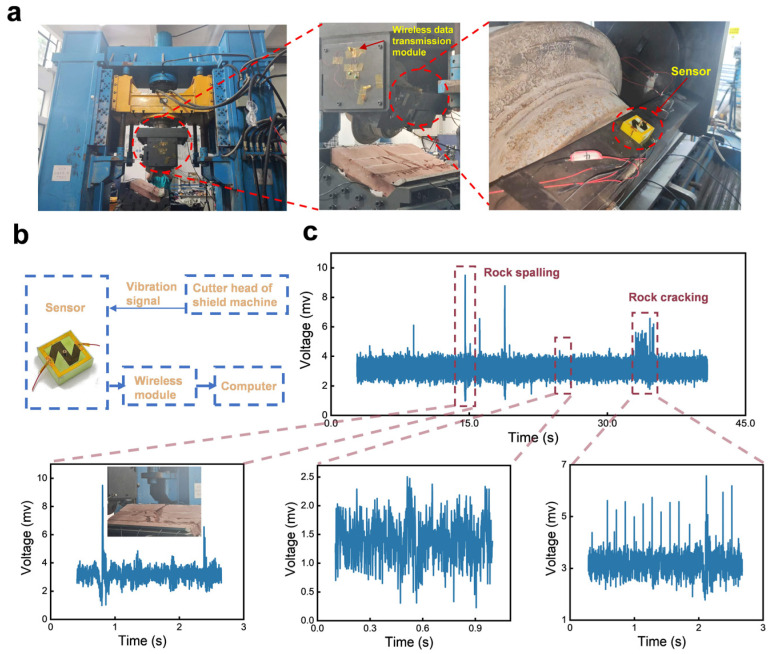
Real-time monitoring of the disc cutter cracking a rock. (**a**) The developed paper-based sensor installed on a shield machine tool experiment platform. (**b**) The test system of the sensor for monitoring the vibrations during the cracking process of rock. (**c**) Real-time signals produced by the sensor when the rock is cut by the disc cutter.

Apart from detecting high-frequency vibrations, the developed paper-based MEMS sensor can also be used to monitor low-frequency vibrations. As shown in Figure 7a, the sensor is installed on the base of a disc cutter, to monitor the vibrations generated by the cutter fracturing rocks. We used a wireless data transmission module to collect the signals from the sensor and transmit the signals to a computer, as the given system chart shows in Figure 7b. Figure 7c shows a complete crushing process of the disc cutter fracturing the rock. Strong peaks occur when the disc cutter makes the rock spall or crack. For example, when the rock is cut by the disc cutter and the surface of the rock begins to spall, large vibrations are generated due to the sudden enhancement of friction and obvious peaks can be found. When the fracture of the disc cutter enters into a stable working state, the real-time responses of the sensor behave stable too. However, once the disc cutter continues to apply friction to the rock, the rock begins to crack more deeply and finally fracture thoroughly. During this period, the peak amplitudes of the sensor are larger than that of uncrack. Therefore, from the output signals of the sensor, the working status of the disc cutter can be reflected by the amplitude and frequency.

## 4. Conclusions

In summary, a paper-based vibration sensor is fabricated by methods of pencil drawing and femtosecond laser. Specifically, the sensor has a squared paper base and a zig-zag-shaped oscillator with a mass in the center. Microgrooves perpendicular to the direction of current as well as the micro/nano cracks formed on the paper surface contribute to the significant electromechanical signal conversion function of the sensor. The results demonstrate that the developed sensor can detect vibration with a frequency ranging from 5 Hz to 1000 Hz, and vibration with acceleration ranging from 1 g to 10 g. The sensor can be utilized for monitoring vibrations generated by knuckle-knocking a plastic plate or objects falling. Also, the sensor can be used to detect vibrations in areas such as the shield cut head to assess the working conditions of machinery. Compared to traditional rigid MEMS vibration sensors, the paper-based MEMS vibration sensor exhibits merits like easy fabrication, low cost, and being environmentally friendly, which indicates its great application potential in vibration monitoring fields.

## Data Availability

The original contributions presented in the study are included in the article, further inquiries can be directed to the corresponding author.

## References

[1] Schöner M.G., Simon R., Schöner C.R. (2016). Acoustic Communication in Plant–Animal Interactions. Curr. Opin. Plant Biol..

[2] Stölting H., Moore T.E., Lakes-Harlan R. (2002). Substrate Vibrations during Acoustic Signalling in the Cicada Okanagana Rimosa. J. Insect Sci..

[3] Tsubaki R., Hosoda N., Kitajima H., Takanashi T. (2014). Substrate-Borne Vibrations Induce Behavioral Responses in the Leaf-Dwelling Cerambycid, *Paraglenea fortunei*. Zool. Sci..

[4] Lee S., Kim J., Yun I., Bae G.Y., Kim D., Park S., Yi I.-M., Moon W., Chung Y., Cho K. (2019). An Ultrathin Conformable Vibration-Responsive Electronic Skin for Quantitative Vocal Recognition. Nat. Commun..

[5] Chu T., Nguyen T., Yoo H., Wang J. (2024). A Review of Vibration Analysis and Its Applications. Heliyon.

[6] Tahmasbi D., Shirali H., Sajad Mousavi Nejad Souq S., Eslampanah M. (2024). Diagnosis and Root Cause Analysis of Bearing Failure Using Vibration Analysis Techniques. Eng. Fail. Anal..

[7] Mortimer B., Soler A., Siviour C.R., Vollrath F. (2018). Remote Monitoring of Vibrational Information in Spider Webs. Sci. Nat..

[8] Chen Y., Wang Y., Zhang Y., Zou H., Lin Z., Zhang G., Zou C., Wang Z.L. (2018). Elastic-Beam Triboelectric Nanogenerator for High-Performance Multifunctional Applications: Sensitive Scale, Acceleration/Force/Vibration Sensor, and Intelligent Keyboard. Adv. Energy Mater..

[9] Luo X., Liu L., Wang Y., Li J., Berbille A., Zhu L., Wang Z.L. (2022). Tribovoltaic Nanogenerators Based on MXene–Silicon Heterojunctions for Highly Stable Self-Powered Speed, Displacement, Tension, Oscillation Angle, and Vibration Sensors. Adv. Funct. Mater..

[10] Shan X., Tang L., Wen H., Martinek R., Smulko J. (2020). Analysis of Vibration and Acoustic Signals for Noncontact Measurement of Engine Rotation Speed. Sensors.

[11] Shen A., Cui W., Zhou J., Cai F. (2024). Effect of Turbine’s Torque and Speed Variation on Hydraulic Vibration Analysis during Transient Processes. Energy Sci. Eng..

[12] Zhai Y., Li H., Tao Z., Cao X., Yang C., Che Z., Xu T. (2022). Design, Fabrication and Test of a Bulk SiC MEMS Accelerometer. Microelectron. Eng..

[13] Ye Y., Wan S., Li S., Peng Y., He X., Qin M. (2024). Fabrication and Characterization of a MEMS Thermal Convective Accelerometer on Silicon-in-Glass Substrate. IEEE Sens. J..

[14] Babatain W., Bhattacharjee S., Hussain A.M., Hussain M.M. (2021). Acceleration Sensors: Sensing Mechanisms, Emerging Fabrication Strategies, Materials, and Applications. ACS Appl. Electron. Mater..

[15] Lee M.-K., Kim B.-H., Lee G.-J. (2023). Lead-Free Piezoelectric Acceleration Sensor Built Using a (K,Na)NbO_3_ Bulk Ceramic Modified by Bi-Based Perovskites. Sensors.

[16] Lee M.-K., Han S.-H., Park K.-H., Park J.-J., Kim W.-W., Hwang W.-J., Lee G.-J. (2019). Design Optimization of Bulk Piezoelectric Acceleration Sensor for Enhanced Performance. Sensors.

[17] Han X., Huang M., Wu Z., Gao Y., Xia Y., Yang P., Fan S., Lu X., Yang X., Liang L. (2023). Advances in High-Performance MEMS Pressure Sensors: Design, Fabrication, and Packaging. Microsyst. Nanoeng..

[18] Song P., Ma Z., Ma J., Yang L., Wei J., Zhao Y., Zhang M., Yang F., Wang X. (2020). Recent Progress of Miniature MEMS Pressure Sensors. Micromachines.

[19] Hajare R., Reddy V., Srikanth R. (2022). MEMS Based Sensors—A Comprehensive Review of Commonly Used Fabrication Techniques. Mater. Today Proc..

[20] Ejeian F., Azadi S., Razmjou A., Orooji Y., Kottapalli A., Ebrahimi Warkiani M., Asadnia M. (2019). Design and Applications of MEMS Flow Sensors: A Review. Sens. Actuators A Phys..

[21] Zhu J., Liu X., Shi Q., He T., Sun Z., Guo X., Liu W., Sulaiman O.B., Dong B., Lee C. (2019). Development Trends and Perspectives of Future Sensors and MEMS/NEMS. Micromachines.

[22] Liu H.-F., Luo Z.-C., Hu Z.-K., Yang S.-Q., Tu L.-C., Zhou Z.-B., Kraft M. (2022). A Review of High-Performance MEMS Sensors for Resource Exploration and Geophysical Applications. Pet. Sci..

[23] Ullo S.L., Sinha G.R. (2020). Advances in Smart Environment Monitoring Systems Using IoT and Sensors. Sensors.

[24] Kaidarova A., Geraldi N.R., Wilson R.P., Kosel J., Meekan M.G., Eguíluz V.M., Hussain M.M., Shamim A., Liao H., Srivastava M. (2023). Wearable Sensors for Monitoring Marine Environments and Their Inhabitants. Nat. Biotechnol..

[25] Park J., Seo B., Jeong Y., Park I. (2024). A Review of Recent Advancements in Sensor-Integrated Medical Tools. Adv. Sci..

[26] Lan L., Ping J., Xiong J., Ying Y. (2022). Sustainable Natural Bio-Origin Materials for Future Flexible Devices. Adv. Sci..

[27] Shahabuddin M., Uddin M.N., Chowdhury J.I., Ahmed S.F., Uddin M.N., Mofijur M., Uddin M.A. (2023). A Review of the Recent Development, Challenges, and Opportunities of Electronic Waste (e-Waste). Int. J. Environ. Sci. Technol..

[28] Pershaanaa M., Bashir S., Kumar S.S.A., Ramesh S., Ramesh K. (2024). Keystones of Green Smart City-Framework, e-Waste, and Their Impact on the Environment—A Review. Ionics.

[29] Shaaban M., Wang X.-L., Song P., Hou X., Wei Z. (2024). Microplastic Pollution and E-Waste: Unraveling Sources, Mechanisms, and Impacts across Environments. Curr. Opin. Green Sustain. Chem..

[30] Zarei M., Lee G., Lee S.G., Cho K. (2023). Advances in Biodegradable Electronic Skin: Material Progress and Recent Applications in Sensing, Robotics, and Human–Machine Interfaces. Adv. Mater..

[31] Kumi M., Wang T., Ejeromedoghene O., Wang J., Li P., Huang W. (2024). Exploring the Potentials of Chitin and Chitosan-Based Bioinks for 3D-Printing of Flexible Electronics: The Future of Sustainable Bioelectronics. Small Methods.

[32] He Y., Zhou M., Mahmoud M.H.H., Lu X., He G., Zhang L., Huang M., Elnaggar A.Y., Lei Q., Liu H. (2022). Multifunctional Wearable Strain/Pressure Sensor Based on Conductive Carbon Nanotubes/Silk Nonwoven Fabric with High Durability and Low Detection Limit. Adv. Compos. Hybrid Mater..

[33] Hui Z., Zhang L., Ren G., Sun G., Yu H., Huang W. (2023). Green Flexible Electronics: Natural Materials, Fabrication, and Applications. Adv. Mater..

[34] Shen L., Zhou S., Gu B., Wang S., Wang S. (2023). Highly Sensitive Strain Sensor Fabricated by Direct Laser Writing on Lignin Paper with Strain Engineering. Adv. Eng. Mater..

[35] Hasnain M., Ullah Z., Sonil N.I., Ahmad W., Khalil A., Ali S.M., Mustafa G.M., Nazar M.F., Rouf S.A., Shamain N. (2023). Ultrasensitive Strain Sensor Based on Graphite Coated Fibrous Frameworks for Security Applications. Mater. Today Commun..

[36] Qi X., Li X., Jo H., Sideeq Bhat K., Kim S., An J., Kang J.-W., Lim S. (2020). Mulberry Paper-Based Graphene Strain Sensor for Wearable Electronics with High Mechanical Strength. Sens. Actuators A Phys..

[37] Liao X., Zhang Z., Liao Q., Liang Q., Ou Y., Xu M., Li M., Zhang G., Zhang Y. (2016). Flexible and Printable Paper-Based Strain Sensors for Wearable and Large-Area Green Electronics. Nanoscale.

[38] Bu Y., Shen T., Yang W., Yang S., Zhao Y., Liu H., Zheng Y., Liu C., Shen C. (2021). Ultrasensitive Strain Sensor Based on Superhydrophobic Microcracked Conductive Ti_3_C_2_Tx MXene/Paper for Human-Motion Monitoring and E-Skin. Sci. Bull..

[39] Liu H., Wang W., Xiang H., Wu H., Li Z., Zhou H., Huang W. (2022). Paper-Based Flexible Strain and Pressure Sensor with Enhanced Mechanical Strength and Super-Hydrophobicity That Can Work under Water. J. Mater. Chem. C.

[40] Yun T., Du J., Ji X., Tao Y., Cheng Y., Lv Y., Lu J., Wang H. (2023). Waterproof and Ultrasensitive Paper-Based Wearable Strain/Pressure Sensor from Carbon Black/Multilayer Graphene/Carboxymethyl Cellulose Composite. Carbohydr. Polym..

[41] Yao D.-J., Tang Z., Zhang L., Liu Z.-G., Sun Q.-J., Hu S.-C., Liu Q.-X., Tang X.-G., Ouyang J. (2021). A Highly Sensitive, Foldable and Wearable Pressure Sensor Based on MXene-Coated Airlaid Paper for Electronic Skin. J. Mater. Chem. C.

[42] Chen S., Song Y., Xu F. (2018). Flexible and Highly Sensitive Resistive Pressure Sensor Based on Carbonized Crepe Paper with Corrugated Structure. ACS Appl. Mater. Interfaces.

[43] Li J., Yao Z., Meng X., Zhang C., Sun T., Song W., Li H., Zhang J., Niu S., Liu L. (2022). Paper-Based Sensor with Bioinspired Macrogrooves for Dual Pressure and Mechanical Strain Signal Detection. ACS Appl. Nano Mater..

[44] Sakhuja N., Kumar R., Katare P., Bhat N. (2022). Structure-Driven, Flexible, Multilayered, Paper-Based Pressure Sensor for Human-Machine Interfacing. ACS Sustain. Chem. Eng..

[45] Guan X., Hou Z., Wu K., Zhao H., Liu S., Fei T., Zhang T. (2021). Flexible Humidity Sensor Based on Modified Cellulose Paper. Sens. Actuators B Chem..

[46] Liu H., Xiang H., Wang Y., Li Z., Qian L., Li P., Ma Y., Zhou H., Huang W. (2019). A Flexible Multimodal Sensor That Detects Strain, Humidity, Temperature, and Pressure with Carbon Black and Reduced Graphene Oxide Hierarchical Composite on Paper. ACS Appl. Mater. Interfaces.

[47] Zhang H., Xia C., Feng G., Fang J. (2021). Hospitals and Laboratories on Paper-Based Sensors: A Mini Review. Sensors.

[48] Immanuel P.N., Huang S.-J., Adityawardhana Y., Yen Y.-K. (2023). A Review of Paper-Based Sensors for Gas, Ion, and Biological Detection. Coatings.

[49] Zhang Z., Liu G., Li Z., Zhang W., Meng Q. (2023). Flexible Tactile Sensors with Biomimetic Microstructures: Mechanisms, Fabrication, and Applications. Adv. Colloid Interface Sci..

[50] Wang M., Lin Z., Ma S., Yu Y., Chen B., Liang Y., Ren L. (2023). Composite Flexible Sensor Based on Bionic Microstructure to Simultaneously Monitor Pressure and Strain. Adv. Healthc. Mater..

[51] Liu L., Jiao Z., Zhang J., Wang Y., Zhang C., Meng X., Jiang X., Niu S., Han Z., Ren L. (2021). Bioinspired, Superhydrophobic, and Paper-Based Strain Sensors for Wearable and Underwater Applications. ACS Appl. Mater. Interfaces.

[52] Liu L., Meng X., Zhang C., Chen Y., Sun T., Lu Z., Zhang J., Niu S., Han Z., Duan J.-A. (2022). A Multifunctional Flexible Sensor with Coupling Bionic Microstructures Inspired by Nature. J. Mater. Chem. C.

[53] Liu D., Zhang H., Chen H., Lee J.-H., Guo F., Shen X., Zheng Q., Kim J.-K. (2022). Wrinkled, Cracked and Bridged Carbon Networks for Highly Sensitive and Stretchable Strain Sensors. Compos. Part A Appl. Sci. Manuf..

